# Functional Trait Responses of *Brasenia schreberi* to Water and Soil Conditions Reveal Its Endangered Status

**DOI:** 10.3390/plants14132072

**Published:** 2025-07-07

**Authors:** Jingyu Yao, Zhenya Liu, Junbao Yu, Yun Zhang, Rui Xu, Jiahua Li, Yang Xu, Mei Sun

**Affiliations:** 1 Yunnan Key Laboratory of Plateau Wetland Conservation, Restoration and Ecological Services, Southwest Forestry University, Kunming 650224, China; 17787845075@163.com (J.Y.); liuzhenya9969@163.com (Z.L.); yu.junbao@gmail.com (J.Y.); zhangyuncool@163.com (Y.Z.); xuyang@lreis.ac.cn (Y.X.); 2 Dianchi Lake Ecosystem Observation and Research Station of Yunnan Province, Kunming 650228, China; 3 Baoshan Management Bureau of Gaoligong Mountains National Nature Reserve in Yunnan, Baoshan 678000, China; xurui891019@163.com (R.X.); glgsljh@163.com (J.L.)

**Keywords:** *Brasenia schreberi*, plateau wetland, plant functional traits, ecological response, water environment, soil nutrient conditions

## Abstract

[Background] *Brasenia schreberi* is a perennial floating leaf aquatic plant with high ecological protection value and potential for economic development, and thus, its endangered mechanisms are of great concern. The rapid endangerment of this species in modern times may be primarily attributed to the deterioration of water and soil environmental conditions, as its growth relies on high-quality water and soil. [Objective] Exploring the responses of *B. schreberi* to water and soil conditions from the perspective of functional traits is of great significance for understanding its endangered mechanisms and implementing effective conservation strategies. [Methods] This study was conducted in the Tengchong Beihai Wetland, which has the largest natural habitat of *B. schreberi* in China. By measuring the key functional traits of *B. schreberi* and detecting the water and soil parameters at the collecting sites, the responses of these functional traits to the water and soil conditions have been investigated. [Results] (1) The growth status of *B. schreberi* affects the expression of its functional traits. Compared with sporadic distribution, *B. schreberi* in continuous patches have significantly higher stomatal conductance, intercellular CO_2_ concentration, transpiration rate, and vein density, while these plants have significantly smaller leaf area and perimeter. (2) Good water quality directly promotes photosynthetic, morphological, and structural traits. However, high soil carbon, nitrogen, and phosphorus contents can inhibit the photosynthetic rate. The net photosynthetic rate is significantly positively correlated with dissolved oxygen content, pH value, ammonia nitrogen, and nitrate nitrogen contents in the water, as well as the magnesium, zinc, and silicon contents in the soil. In contrast, the net photosynthetic rate is significantly negatively correlated with the total phosphorus content in water and the total carbon, total nitrogen, and total phosphorus content in the soil. (3) Leaf area and perimeter show positive correlations with various water parameters, including the depth, temperature, pH value, dissolved oxygen content, ammonium nitrogen, and nitrate nitrogen content, yet they are negatively correlated with total phosphorus content, chemical oxygen demand, biological oxygen demand, and permanganate index of water. [Conclusions] This study supports the idea that *B. schreberi* thrives in oligotrophic water environments, while the notion that fertile soil is required for its growth still needs to be investigated more thoroughly.

## 1. Introduction

*Brasenia schreberi*, a perennial floating leaf plant in the family Nymphaeaceae, is a relic and rare species from the Tertiary flora, characterized by its submerged structures being covered with mucilage produced by epidermal glandular hairs [1]. The mucilage primarily consists of polysaccharides and is particularly abundant in the tender tissues of *B. schreberi*, such as buds and young stems [2,3]. *B. schreberi* exhibits a distribution primarily confined to subtropical regions below 40° N latitude and at elevations below 2500 m, classifying it as belonging to the group of thermophilic aquatic species [4,5]. As a pioneer species in freshwater ecosystems, *B. schreberi* often becomes the dominant species in water deeper than the growth limit of emergent aquatic plants, due to its strong asexual reproduction ability and the inhibitory effect of the mucilage on other plants [6,7]. This plant is also a traditional edible and medicinal aquatic plant in Asia, especially in China, with its edible parts mainly being the tender buds and stems that have a thick and transparent gelatinous substance, giving it a unique texture [3,8,9]. During the early 20th century, *B. schreberi* exhibited a broad distribution across unpolluted freshwater habitats, including ponds, marshes, lakes, and even agricultural wetlands, spanning Asia, North America, and Australia [1,10]. In recent decades, the suitable aquatic habitats for wild *B. schreberi* have significantly decreased, leading to a dramatic shrinkage in its distribution area and a marked decline in population size and numbers, affected by environmental pollution, overharvesting, and climate change, and as a result, it has been listed as a key protected wild plant species by many countries [1,11]. Understanding the mechanisms of the endangered status of *B. schreberi* is crucial for its scientific conservation and utilization, and conducting research on its adaptability to the growth environment is an important aspect in exploring these mechanisms.

Several studies have explained the reasons for the disappearance of natural *B. schreberi* populations by exploring the adaptability of its phenological, reproductive, morphological, and structural characteristics to the environment. For example, the extinction of *B. schreberi* in Europe during geological periods may have been primarily due to its meristematic tissues, such as winter buds and rhizome apices, being unable to withstand freezing temperatures and thus failing to produce winter buds for overwintering during glacial and interglacial periods [1,12]. The flowers of *B. schreberi* exhibit unisexual sequential blooming (dioecious), a mechanism to avoid self-pollination, but cross-pollination may be hindered due to the reduced number of plants and weakened individuals, which is another potential reason for its endangered status [13]. Anatomical analysis of *B. schreberi* reveals that its tender parts are covered by a fragile barrier of hydrophilic mucilage and a discontinuous cuticle, and it is hypothesized that this makes its epidermal glandular trichomes and the underlying parenchyma cells susceptible to high ionic strength and organic pollutants in contaminated water, damaging the plant’s barrier tissues and preventing normal growth and development, thus leading to its endangerment [10,14,15]. In summary, the findings indicate that over large temporal and spatial scales, climate changes (e.g., temperature) and reproductive strategies may significantly impact *B. schreberi*, while the rapid contemporary endangerment of *B. schreberi* is closely linked to water and soil conditions.

*B. Schreberi* is adapted to oligotrophic aquatic environments, requiring clean, flowing water for growth, habitat, and water environment protection are crucial for maintaining its populations [1,16,17]. *B. schreberi* thrives best in waters with near-neutral pH, low conductivity, and low turbidity [18,19]. Small amounts of fertilizer and pesticides that are discharged into the water in the field can cause the leaves of *B. schreberi* to rot within a few days [20]. Low levels of dissolved oxygen in water will have specific negative impacts on the growth of *B. schreberi* [21]. As residue and COD in water increase, *B. schreberi* shows more decayed leaves and black roots, changes from dark green to grayish green, and declines from vigorous to slender growth or even die off [22]. The permanganate index, total N content, and conductivity of water are all negatively correlated with the mucilage components, thickness, and single bud weight of *B. schreberi* [17]. Studies on the artificial propagation of *B. schreberi* have also indicated that, in addition to good water quality, *B. schreberi* requires soil rich in organic matter content for its growth. *Brasenia schreberi* grows best in slightly acidic paddy soils with high organic matter and total nitrogen, adequate nitrogen supply, deep soil, good surface soil structure, soft soil body, and a thick mud layer [22]. Applying chemical and organic fertilizers in soil can boost the growth of *B. schreberi* and enhance its yield and quality [23]. The mucilage content and individual bud weight of *B. schreberi* are significantly positively correlated with the organic matter and total nitrogen content of the sediment [17]. These studies provide some explanations for the ecological adaptation mechanisms of *B. schreberi*. However, they mainly focus on growth performance and mucilage characteristics to explore the environmental impacts on it, with limited research on other morphological, structural, and photosynthetic physiological parameters closely related to resource capture, growth rate, ecological strategies, and environmental responses. Intensifying research on these parameters under water and soil microenvironment conditions can more directly explain the growth status and endangerment mechanisms of *B. schreberi*.

China has the largest scale and longest history of cultivating and using *B. schreberi*. In China, it mainly occurs in provinces south of the Yangtze River, growing in nature reserves with good natural environments. Tengchong Beihai Wetland is one of the most important areas for wild *B. schreberi* in China. In recent years, local departments have implemented projects such as afforestation around wetlands, integrated protection and management, and biodiversity conservation and restoration. These initiatives have led to an increase in the wild *B. schreberi* population in Tengchong Beihai Wetland, expanding its distribution area from 0.2 ha to over 100 ha, which now exceeds the combined area of other provinces. This study examines wild *B. schreberi* in the Tengchong Beihai Wetland Provincial Nature Reserve, Yunnan, China, measuring its photosynthetic and morphological parameters to explore its physiological and ecological responses to water and soil conditions from a physiological ecology perspective. This study hypothesizes that *Brasenia schreberi* growth correlates positively with good water quality parameters (e.g., dissolved oxygen, pH) and negatively with water pollution indicators (e.g., total nitrogen, total phosphorus, BOD, COD). Additionally, based on the experience of artificial propagation of *Brasenia schreberi*, its growth also requires moderately fertile soil. However, this conclusion still needs to be verified by specific experiments and may lead to different conclusions due to the differences in soil nutrient levels of different wetlands. This study will investigate the responses of *B. schreberi* to water and soil from a physiological and ecological perspective, and explore its mechanisms of endangerment.

## 2. Results

### 2.1. Differences in Functional Traits of Brasenia schreberi Between Two Clustering Groups

**Figure 1:**
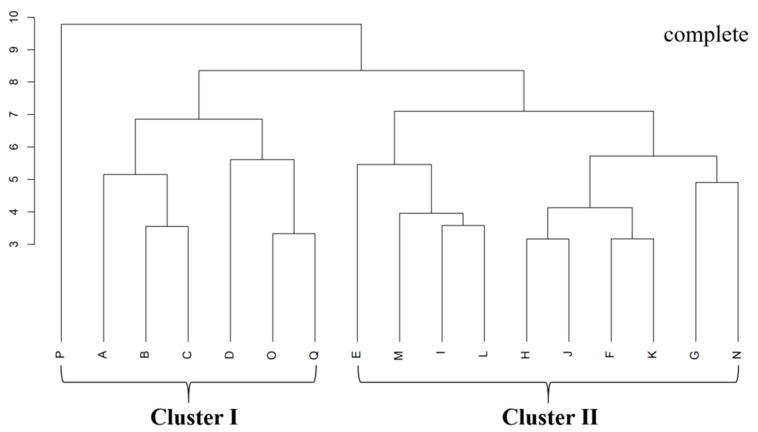
Cluster diagram of *Brasenia schreberi* based on the functional traits. The vertical axis values represent the Euclidean distance. Letters A~Q are sampling points, Cluster I is a *B. schreberi* community with sporadic distribution, and Cluster II is a *B. schreberi* community with continuous distribution.

**Figure 2:**
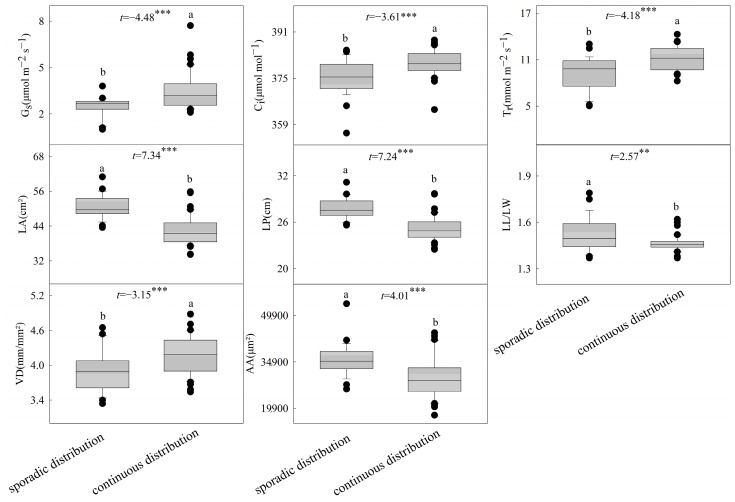
Differences in functional traits between the two cluster groups in *Brasenia schreberi*. P_n_, net photosynthetic rate; G_s_, stomatal conductance; C_i_, intercellular carbon dioxide concentration; T_r_, transpiration rate; LA, leaf area; LP, leaf perimeter; L/W, ratio of leaf length to width; LP^2^/LA, ratio of square of leaf perimeter to area; SD, stomatal density; SL, stomatal length; SW, stomatal width; SA, stomatal area; VD, leaf vein density; BA, vascular bundle area; AS, air space area; AA, spiracle area; CT, cuticle thickness; ET, epidermal thickness. **, *p* < 0.01; ***, *p* < 0.001.

The clustering results of *B. schreberi*’s functional traits are in line with its natural growth conditions. Cluster I sampling sites have sparsely distributed *B. schreberi*, while Cluster II sites have densely distributed plants (Figure 1), showing that the functional traits of *B. schreberi* are affected by its growth status. Compared to sparsely distributed *B. schreberi*, clustered-distribution *B. schreberi* has significantly higher stomatal conductance, intercellular CO_2_ concentration, transpiration rate, and vein density (*p* < 0.05); yet, its leaf area, perimeter, length to width ratio, air cavity area, and aerenchyma area are significantly smaller (*p* < 0.05; Figure 2). No significant differences were observed in other functional traits between groups. These results indicate that the different growth statuses of *B. schreberi* may influence its physiological and morphological traits as adaptive responses to environmental changes.

### 2.2. Ecological Response of Functional Traits of Brasenia schreberi to Water and Soil Environmental Factors

The water environment and soil nutrient parameters significantly influence the functional trait expression of *B. schreberi*. Random forest analysis revealed that most aquatic parameters (WH, WT, NH_4_^+^, NO_3_^−^, COD_Mn_, COD, and N_water_) affected photosynthetic traits, while the primary soil factors influencing photosynthesis were P_soil_, K, and Zn (Figure S2). Among them, the main parameters affecting P_n_ were three water environmental parameters (COD, WH, and COD_Mn_), the main parameters affecting G_s_ were P_soil_, WH, and N_water_, the main parameters affecting C_i_ were NO_3_^−^, K, and Zn, and the main parameters affecting T_r_ were WT, P_soil_, and NH_4_^+^ (Figure S2).

The parameters affecting the size and shape of *B. schreberi* leaves were mainly DO, WT, WH, NH_4_^+^, P_soil_, N_soil_, and Ca (Figure S2). Among them, LA was mainly affected by DO, WT, and WH; LP was mainly affected by DO, WT, and Ca; L/W was mainly affected by WH, DO, and P_soil_; and LP^2^/LA was mainly affected by pH, N_soil_, and NH_4_^+^ (Figure S2).

The main water environment parameters affecting stomata were DO, NH_4_^+^, and pH, and the main soil nutrient parameters affecting stomata were N_soil_, P_soil_, and Ca (Figure S2). Among them, the main parameters affecting SD were pH and N_soil_ and P_soil_; those affecting SL were DO, P_soil_, and Ca; those affecting SW were NH_4_^+^, DO, and P_soil_; and those affecting SA were NH_4_^+^, N_soil_, and P_soil_ (Figure S2).

The water environment factors affecting the anatomical structural traits of *B. schreberi* were mainly WT, DO, WH, pH, and P_water_, while the soil factors affecting these functional traits were mainly P_soil_ and trace elements, including Si, Zn, Mg, and K (Figure S2), suggesting that the structural trait parameters were more susceptible to the influence of soil trace elements. Among them, WT, DO, and Si were the main parameters affecting VD; WT, pH, and DO were the main parameters affecting BA; WH, K, and Zn were the main parameters affecting AS; WT, DO, and Si were the main parameters affecting AA; WT, Mg, and P_soil_ were the main parameters affecting CT; and P_water_, NH_4_^+^, and K were the main parameters affecting ET (Figure S2).

Overall, the water environmental parameters of water depth, water temperature, dissolved oxygen content, ammoniacal nitrogen, pH, N_soil_, and P_soil_ in the substrate parameters had a greater effect on the functional characteristics of *B. schreberi*, while the water pollution indicators of N_water_, COD, and COD_Mn_ mainly affected the photosynthetic parameters, and the soil trace elements mainly affected the anatomical and structural characteristics of *B. schreberi* (Figure S2).

Further bivariate correlation analysis between functional traits and environmental factors showed that *B. schreberi* P_n_ was significantly positively correlated with pH, DO, NH_4_^+^, and NO_3_^−^ in water and Mg, Zn, and Si in soil, while it was significantly negatively correlated with P_water_ in water and C_soil_, N_soil_, and P_soil_ in soil (*p* < 0.05); G_s_ was significantly negatively correlated with N_water_ (*p* < 0.05); T_r_ was significantly positively correlated with BOD, COD, and COD_Mn_ of water and K, Zn, and Si of soil, while it was significantly negatively correlated with WH, WT, C_soil_, N_soil_, and P_soil_ of soil (*p* < 0.05); C_i_ was significantly negatively correlated with pH, DO, NH_4_^+^, and NO_3_^−^ of water, while there were significant positive correlations (*p* < 0.05) between C_i_ and P_water_ and COD of water (Table 1). In the relationship between photosynthetic parameters and environmental factors, the overall trend was consistent among P_n_, G_s_, and T_r_, while their overall relationship with C_i_ was opposite (Table 1).

Leaf size parameters, including leaf area and leaf perimeter, were significantly and positively correlated with WH, WT, pH, DO, NH_4_^+^, and NO_3_^−^, and significantly and negatively correlated with P_water_, BOD, COD, and COD_Mn_ (*p* < 0.05; Table 1). There was also a significant positive correlation between L/W and DO and a significant positive correlation between LP^2^/LA and NH_4_^+^ (*p* < 0.05; Table 1).

Among 190 potential relationships between 10 structural traits and 19 hydroedaphic parameters, only 18 showed statistically significant correlations (Table 1). Among the relationships of these functional traits with water and environmental parameters, significant trait environment correlations were identified: SL showed positive relationships with pH, NH_4_^+^, and NO_3_^−^; SA positively correlated with NO_3_^−^; VD negatively associated with WT, DO, and NO_3_^−^; AA positively linked to DO but negatively to P_water_; CT decreased with WT; ET negatively correlated with P_water_ but positively with NH_4_^+^ (Table 1). Among these functional traits, only stomatal parameters and VD exhibited statistically significant correlations with soil parameters (Table 1); SD showed a significant positive correlation with P_soil_, whereas stomatal size parameters, including SL, SW and SA, showed significant negative correlations with P_soil_; and VD showed a significant positive correlation with soil Ca and a significant negative correlation with Mg and Si (Table 1).

Integrated analysis revealed that hydroedaphic conditions predominantly influenced photosynthetic traits in *B. schreberi*, while morphological parameters were mainly affected by aquatic factors. Anatomical traits showed limited significant correlations with either water or soil parameters (Table 1). Among the abiotic parameters, there was generally a negative correlation between N_water_, P_water_, BOD, COD, and COD_Mn_, which indicate the degree of water pollution, and P_n_, G_s_, T_r_, and leaf size, which indicate the photosynthetic productivity of *B. schreberi*, while there was a positive correlation between these traits and abiotic parameters such as DO, pH, NH_4_^+,^ and NO_3_^−^, which indicate good water quality conditions (Table 1). Meanwhile, there were negative correlations between photosynthetic parameters in general and soil C_soil_, N_soil_, P_soil_, and Ca, while positive correlations were found with soil trace elements such as Mg, Zn, and Si (Table 1).

**Figure 3:**
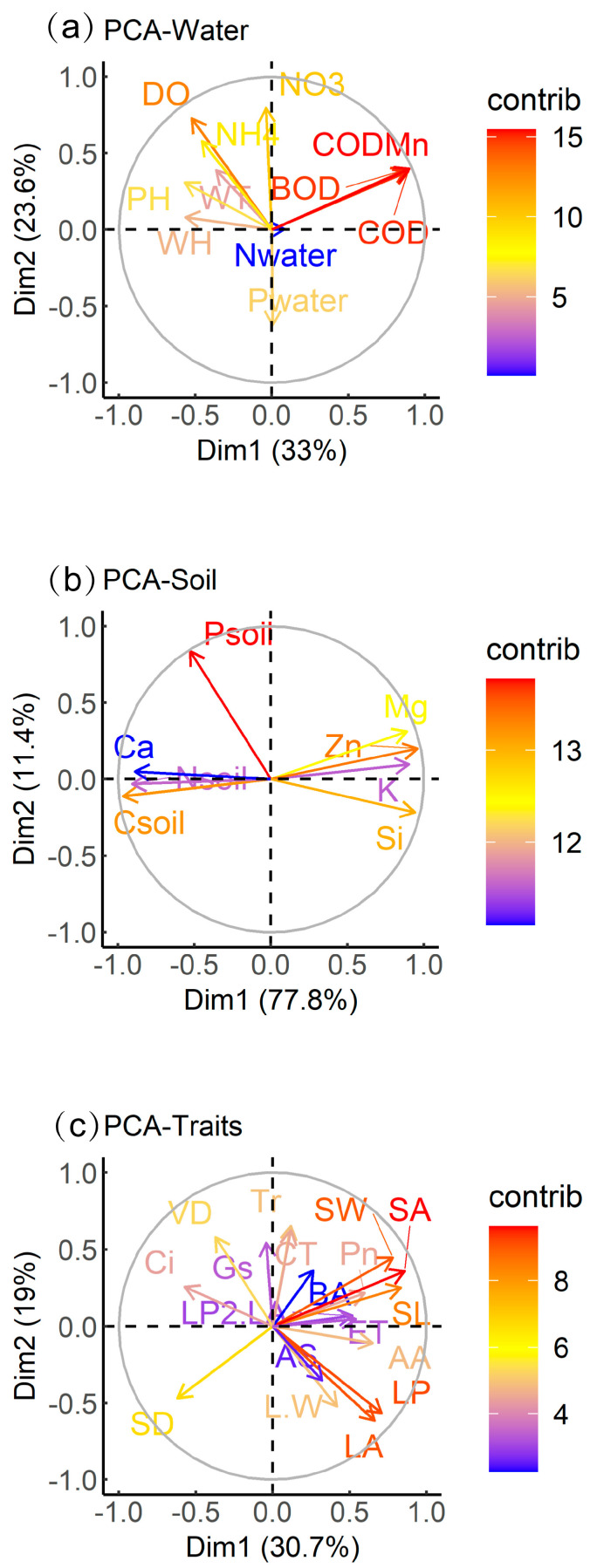
Principal component analysis of plant traits, water factors, and sediment factors. Abbreviations of functional traits and environmental parameters in the figure are consistent with those in Table 1.

Principal component analysis of *B. schreberi*’s functional traits and water and soil factors showed that WH, WT, pH, DO, NH_4_^+^, NO_3_^−^, BOD, COD, and COD_Mn_ contributed more to the total variance, while N_water_ contributed less to the total variance; soil parameters C_soil_, N_soil_, P_soil_, K, Ca, Mg, Zn, and Si all contributed more to the total variance; all functional traits were far from the origin and contributed more to the total variance (Figure 3).

**Figure 4:**
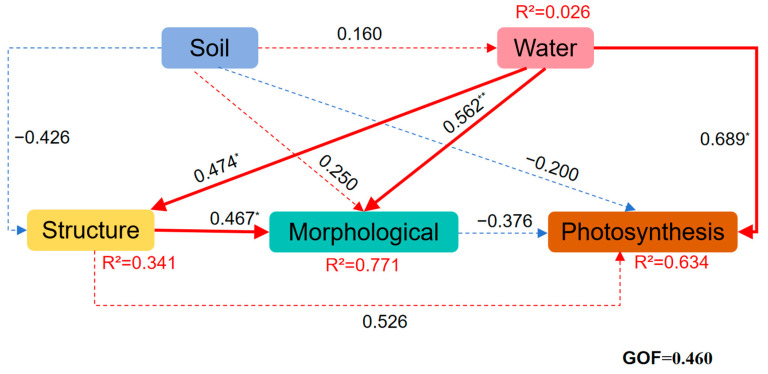
Partial least squares path model (PLS-PM) of plant functional traits and environmental factors. Solid red and blue arrows indicate positive and negative relationships; the numbers on the arrows are normalized path coefficients. The model performance was assessed using a goodness-of-fit (GOF) index. *, *p* < 0.05; **, *p* < 0.01.

Based on the functional traits and environmental parameters selected by PCA, we further constructed a structural equation model to explore the causal relationship between the functional traits of *B. schreberi* and water and soil factors. Applied partial least squares path modeling revealed the different pathways influencing the functional traits of *B. schreberi* (Figure 4). Water and soil factors directly influence the photosynthetic, morphological, and structural traits of *B. schreberi*. Water quality parameters significantly promote its morphological, structural, and other traits (*p* < 0.05), while soil nutrient conditions somewhat inhibit its structural and photosynthetic traits (Figure 4). By integrating random forest models and “trait environment” bivariate correlations, it is suggested that parameters representing good water quality conditions (e.g., DO, WH, WT, pH, NH_4_^+^, NO_3_^−^) play a more prominent role in indicating water environment conditions. These water environment parameters, by influencing the morphology and structure of *B. schreberi*, promote its photosynthetic productivity and leaf size. Among soil parameters, soil nutrient content (C_soil_, N_soil_, and P_soil_) plays a more prominent role in the index of soil nutrient conditions. When these soil nutrient levels are too high, they can limit the structural traits of *B. schreberi*, which in turn can be detrimental to its photosynthetic productivity and leaf expansion.

### 2.3. Correlations Between Functional Traits in Brasenia schreberi Leaves

**Figure 5:**
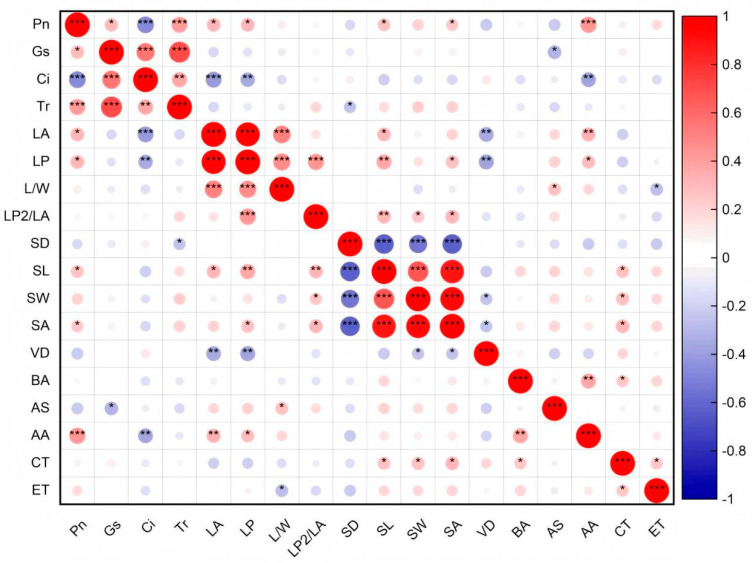
Correlations among leaf functional traits of *Brasenia schreberi*. *, *p* < 0.05; **, *p* < 0.01; ***, *p* < 0.001. P_n_, net photosynthetic rate; G_s_, stomatal conductance; C_i_, intercellular carbon dioxide concentration; T_r_, transpiration rate; LA, leaf area; LP, leaf perimeter; L/W, ratio of leaf length to width; LP^2^/LA, ratio of square of leaf perimeter to area; SD, stomatal density; SL, stomatal length; SW, stomatal width; SA, stomatal area; VD, leaf vein density; BA, vascular bundle area; AS, air space area; AA, spiracle area; CT, cuticle thickness; ET, epidermal thickness.

During the adaptation of *B. schreberi* to water and soil conditions, there are functional linkages among different traits, manifested as significant correlations between traits (Figure 5).

**Figure 6:**
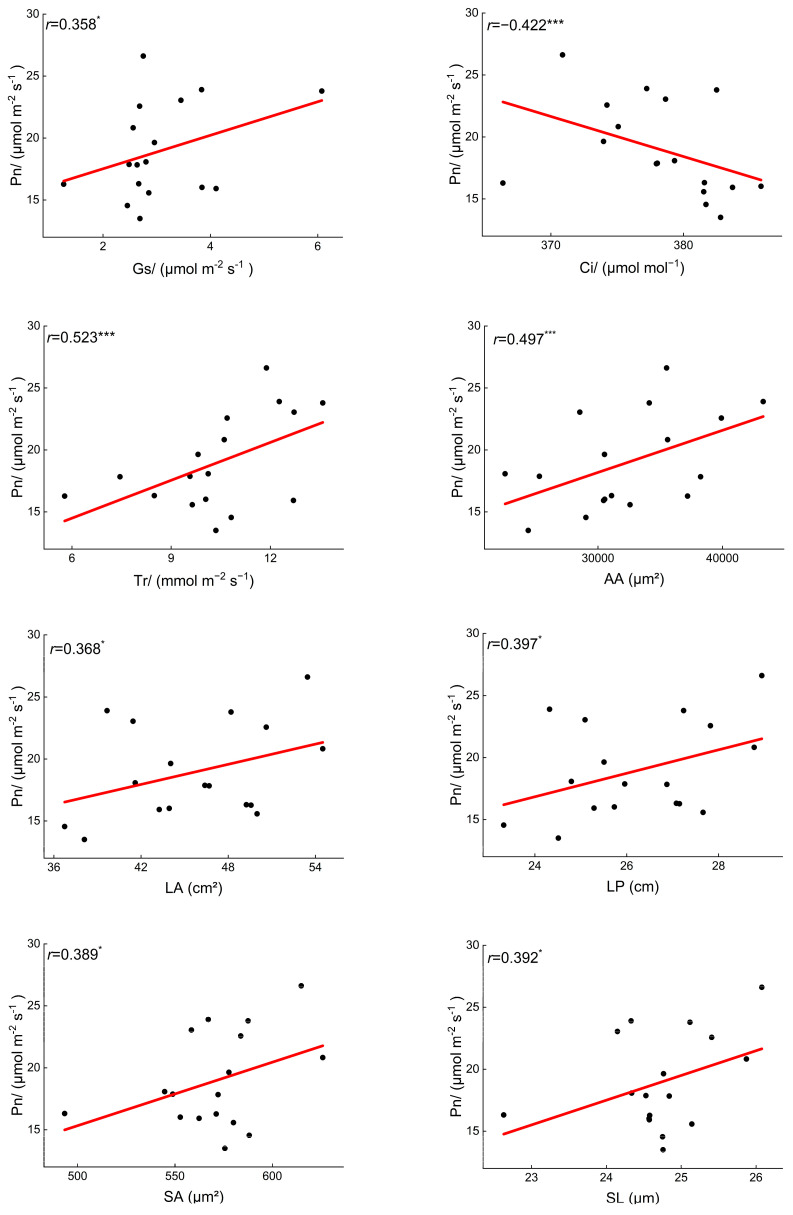
Significant correlations between net photosynthetic rate and other leaf functional traits. P_n_, net photosynthetic rate; G_s_, stomatal conductance; C_i_, intercellular carbon dioxide concentration; T_r_, transpiration rate; LA, leaf area; LP, leaf perimeter; SL, stomatal length; SA, stomatal area. *, *p* < 0.05; ***, *p* < 0.001.

Overall, the structural traits of *B. schreberi* significantly influence its morphological traits, and together they affect its photosynthetic performance. (*p* < 0.05; Figure 4). Specifically, there were significant positive correlations (*p* < 0.05) between P_n_ and G_s_, T_r_, LA, LP, and stomatal size (Figure 6), indicating that there was a positive correlation between *B. schreberi*’s photosynthetic carbon assimilation rate and stomatal water and CO_2_ exchange capacity and leaf light capture capacity, and a negative correlation between stomatal sensitivity and stomatal and leaf vein transport efficiency.

Leaf size parameters, including leaf area and perimeter, show a significant positive correlation with stomatal length and stomatal area, and a significant negative correlation with vein density. (*p* < 0.05; Figure 5). The vascular bundle area, stomatal area, and cuticle thickness are positively correlated (Figure 5), indicating a coordinated role in the transport capacity of vascular bundles, air transmission via stomata, and the barrier function of the cuticle.

## 3. Research Area and Methods

### 3.1. Study Area Overview

**Figure 7:**
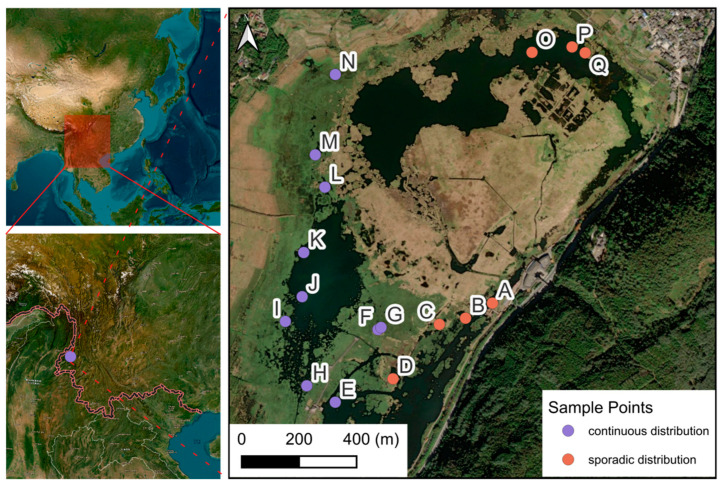
Research sites of *Brasenia schreberi* in Tengchong Beihai Wetland. A–Q, sampling point number; red dots, sporadic distribution; purple dots, continuous distribution.

The research was conducted in Beihai Wetland Provincial Nature Reserve (25°06′42″–25°08′49″ N, 98°30′55″–98°35′02″ E), a protected volcanic wetland ecosystem in Tengchong, Yunnan Province, China (Figure 7). Beihai Wetland, a volcanic dam lake formed by andesite lava flows blocking the Daying River Valley during the Quaternary Period, is located at the southwest edge of the northern subtropics. Its rainfall is influenced by both the Pacific winter monsoon and the Indian Ocean summer monsoon. According to Tengchong Weather Station, the area has an average annual temperature of 14.7 °C, average annual precipitation of 1527.1 mm, evaporation of 1591.3 mm, relative humidity of 79%, drought index of 0.2–0.75, prevailing southwest winds, and wind speeds of 1.6–3.5 m/s.

The long-term water level elevation of Beihai Wetland is 1730 m, and its water surface area is about 200 hm^2^. According to the background value research report of Beihai Wetland water quality from 2022 to 2024, Beihai Wetland has clear water and good water environment quality, generally belonging to Class II water, with some sites being Class III water. Its eutrophication level ranges from mild to moderate. The lake supports abundant submerged vegetation (*Utricularia vulgaris*, *Hydrilla verticillata*, *Myriophyllum spicatum*, *Ottelia acuminata*) and floating leaved plants (*Brasenia schreberi*, *Trapa maximowiczii Korsdh*), indicating superior water quality.

Currently, Beihai Wetland is mainly divided into two parts: the north and the south. The northern part, the original Beihai Wetland, has an average water depth of 3 m and a maximum depth of 7 m. Within this area, *Brasenia schreberi* is sparsely distributed in regions where the water depth is less than 3 m, while in the northeast, there are extensive areas of marshy floating-mat-type meadows. The southern part was originally paddy fields reclaimed by farmers along the lakeside and has fertile soil. It was restored to wetland by the local government through a “farmland to wetland” project in 2013. At present, the water depth in the southern part is 0.5–2 m, and there is a large-scale, continuous distribution of *Brasenia schreberi*, with a cover of 100%. The southern reclaimed wetland area is also the main distribution area of *Brasenia schreberi* in Beihai Wetland. Based on the comparison between the two parts, the water depth in the northern part is deeper, while the soil in the southern part is more fertile. The water environment does not differ significantly between the two parts.

### 3.2. Research Materials

**Figure 8:**
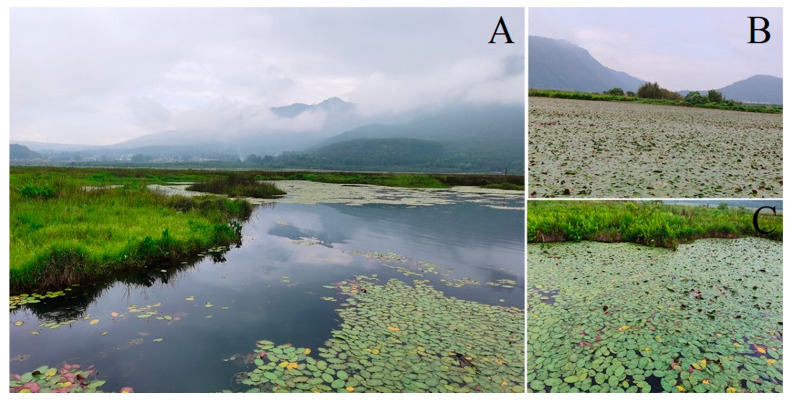
The landscape and its natural communities of *Brasenia schreberi* in Tengchong Beihai wetland. (**A**) The landscape of Tengchong Beihai Wetland; (**B**) *B. schreberi* is densely distributed in the southern and southwestern parts of the Tengchong Beihai Wetland; (**C**) *B. schreberi* is sporadically distributed in the northern and eastern parts of the Tengchong Beihai Wetland.

In this study, the natural *B. schreberi* in Beihai Wetland in Tengchong, Western Yunnan, was used as the research object. According to the field survey, *B. schreberi* in Beihai Wetland mainly showed two growth states, one was *B. schreberi* growing in the northern part of Beihai Wetland, which was sporadically distributed (Figure 8A,C), and the other was *B. schreberi* growing in the southwestern part of Beihai Wetland and the southern part of the hiking trail, which was in a large continuous and overlapping distribution (Figure 8B). In this study, based on the growth condition of *B. schreberi*, 17 study sites were evenly selected in the distribution area of *B. schreberi* in Beihai Wetland, of which 7 study sites were sporadically distributed and 10 study sites had a large continuous and overlapping distribution (Figure 7). Since the propagation strategy of its underground stems, the selection of samples should try to keep each repeat at more than 5 m, after thorough investigation and consultation with the managers and manufacturers of artificially cultivated *Brasenia schreberi*. Therefore, this study selected five healthy and similarly growing plants at each study site, with a minimum of 5 m between each plant to prevent the interference of asexual reproduction in *B. schreberi*.

### 3.3. Measurement of Plant Traits

Photosynthetic parameters of *B. schreberi* were measured during the peak growth period of *B. schreberi* from June to July 2022–2023, with daily measurements from 9:00 a.m. to 12:00 a.m., and the determination of photosynthetic parameters at all sampling sites was completed within one week. Net photosynthetic rate (P_n_, μmol m^−2^ s^−1^), stomatal conductance (G_s_, mol m^−2^ s^−1^), intercellular CO_2_ concentration (C_i_, μmol mol^−1^), and transpiration rate (T_r_, mmol m^−2^ s^−1^) were measured on healthy mature leaves using an LI-6800 photosynthetically active fluorescence meter (LI-6800, LICOR, Lincoln, NE, USA) under field conditions. Prior to measurements, a small CO_2_ bottle was installed, and the instrument was warmed up for 30 min. The CO_2_ concentration in the leaf chamber was adjusted to 400 μmol mol^−1^. The temperature of the leaf chamber (25–27 °C) and the relative humidity of the air (75–80%) were kept at a natural level. For measurement, *B. schreberi* were first induced with 1800 μmol m^−2^ s^−1^ light for 2 min to maintain maximum stomatal conductance, and then the light intensity was adjusted to 1500 μmol m^−2^ s^−1^, and the CO_2_ concentration in the leaf chamber was allowed to equilibrate. The photosynthetic parameters of each plant were measured three times, and the average value was taken as the photosynthetic value for one replicate. The average value of all replicates at each sampling site was used as the photosynthetic value for that site.

**Figure 9:**
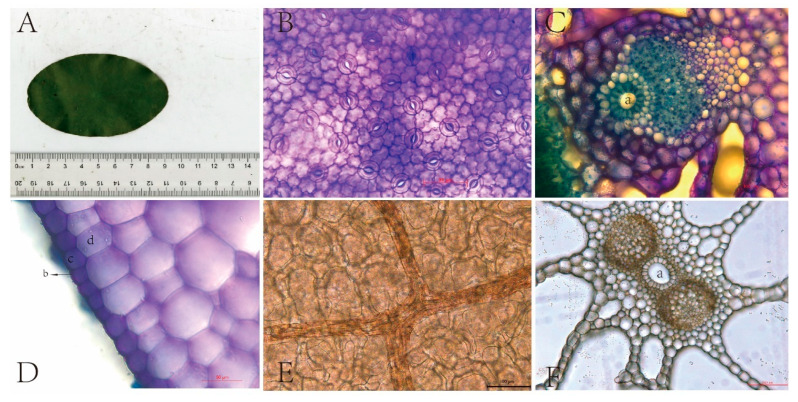
Anatomical structure of *Brasenia schreberi.* (**A**) leaf morphology; (**B**) stomata; (**C**) vascular bundle structure; (**D**) epidermal structure; (**E**) vein; (**F**) air hole. a, air cavity; b, cuticle; c, epidermic cell; d, mesophyll cell.

In the laboratory, after the determination of photosynthetic parameters, *B. schreberi* leaves were scanned in plan view with an 11000XL (Epson Expression) flatbed scanner in the presence of a ruler (Figure 9A) and then manually measured with ImageJ (https://imagej.net/contribute/citing) (accessed on 21 March 2024) [24] for leaf length (LL, μm), width (LW, μm), perimeter (LP, μm), and area (LA, μm^2^), and other morphological parameters were measured manually using ImageJ. Leaf shape index is an important index to measure the morphology of plant leaves and their degree of variability. In this study, the ratio of leaf length to width (L/W) and the ratio of leaf perimeter squared to leaf area (LP^2^/LA) were calculated as leaf shape index.

After scanning the leaves, the centre of one side (approximately 8 mm × 6 mm) was cut for freehand transverse sectioning, and the remainder of the leaves were placed in centrifuge tubes filled with 50% alcohol. Sections were dehydrated, stained, and diluted to produce temporary water slides, which were placed under a Leica microscope (Leica Corp DM6B, Germany) to observe and photograph leaf vascular structures (400×), leaf cuticle and epidermal cells (400×), and leaf apertures (100×) (Figure 9 and Figure S1). Leaf vascular bundle area (BA, μm^2^), air aperture area (AA, μm^2^), air space area (AS, μm^2^), cuticle thickness (CT, μm), and epidermal thickness (ET, μm) were measured and counted using Image J software (https://imagej.net/contribute/citing).

The other half of the blades were removed from the centrifuge tube, and the appropriate size of the two blades was placed in a weighing bottle (40 × 25), with a mass fraction of 25% sodium hydroxide immersion. The solution was changed every 2 days, and the samples were subjected to continuous immersion for one week. After the colour of the sodium hydroxide solution had stopped changing, the sample was rinsed 5 times with distilled water and then soaked in distilled water for 30 min. The distilled water was then poured off and replaced with graded concentrations of alcohol (25%, 50%, 75%, 98%) for 30 min each time.

For the determination, the leaves were removed and placed on two microscope slides, one with the upper epidermis facing up to observe the stomata and the other with the lower epidermis facing up to observe the veins. The leaves were then stained with 1% toluidine blue solution, and after one minute the leaves and slides were lightly rinsed with 50% alcohol to remove excess toluidine blue solution, and then the excess solution around the slide was absorbed with absorbent paper and a temporary water slide was made and photographed under a light microscope. Stomata were observed and clear pictures of stomata were taken at 400× magnification (Figure 9 and Figure S1); leaf veins were observed and clear pictures of leaf veins were taken at 200× magnification (Figure 9 and Figure S1). Stomatal length (SL, μm), stomatal width (SW, μm), and stomatal area (SA, μm^2^) were later measured and recorded using Image J software, and the number of stomata was counted. Stomatal density (SD, no./mm^2^) was counted as the number of stomata per unit leaf area, and vein density (VD, mm/mm^2^) was counted as the total length of veins per unit area. Five plants were selected from each sampling site, and six values for each trait were counted for each plant to ensure that 30 values were counted for each trait at each study site.

### 3.4. Determination of Soil Elemental Content

After measuring the photosynthetic parameters of *Brasenia schreberi*, soil samples from each sampling site were collected immediately to ensure the consistency of sampling time. Soil samples of 1 kg were collected at each sampling point using a fixed-depth peat auger (Eikel Kampala 0423SA, The Netherlands). The samples were then returned to the laboratory for natural drying. After air drying, samples were ground using a soil grinder and sieved through a 100 mesh sieve, then sealed for storage. These soil samples were sent to a third-party testing organisation at the Xishuangbanna Tropical Botanical Garden of the Chinese Academy of Sciences to determine the mass fraction (ω, g kg^−1^) of eight elements, namely carbon (C_soil_), nitrogen (N_soil_), phosphorus (P_soil_), potassium (K), calcium (Ca), magnesium (Mg), zinc (Zn), and silicon (Si), which were counted as the corresponding elemental content.

### 3.5. Determination of Water Parameters

A multi-parameter water quality analyzer (YSI 650 MDS) was used to measure dissolved oxygen (DO, mg L^−1^), pH (pH, mol L^−1^), and water temperature (WT, °C) on site at each sampling point. Water depth (WH, m) was measured by vertically inserting a bamboo pole from the water surface to the substrate and using a tape measure to measure the length of the submerged part. The measurement of the above-mentioned water environment parameters was carried out simultaneously with the measurement of the photosynthetic parameters of *Brasenia schreberi*. Then, 500 mL water samples were collected from each sampling point and transported back to the laboratory. In the laboratory, 40 mL of water from each sampling site was filtered and then analysed using a continuous flow analyser (Germany SEAL Analytical AA3) to determine and calculate the volume fraction of total nitrogen (N_water_, mg L^−1^), total phosphorus (P_water_, mg L^−1^), ammoniacal nitrogen (NH_4_^+^, mg L^−1^), ammonium nitrogen (NO_3_^−^, mg L^−1^), and nitrate nitrogen (NO_3_^−^, mg L ^−1^). The remainder of the water samples were sent to a third-party professional testing organisation for analysis of Biochemical Oxygen Demand (BOD, mg L^−1^), 5-day Chemical Oxygen Demand (COD, mg L^−1^), and Potassium Permanganate Index (COD_Mn_, mg L^−1^).

### 3.6. Data Analysis

Data from this study were analysed using SPSS (v.27, https://spss.en.softonic.com), Canoco (5.0, https://www.canoco5.com) [25] and R (4.3.1, https://cran.r-project.org/src/) (accessed on 16 July 2024) [26] with built-in ‘vegan’ [27] and ‘plspm’ [28] packages. Based on the functional characteristics of *Brasenia schreberi* at 17 sampling points, cluster analysis was performed using the ”complete” method. The results of the cluster analysis were consistent with the distribution of *B. schreberi*, which was significantly clustered into two groups: Group I was the sporadic distribution of points, including 7 sampling points; and Group II was the large area of continuous distribution of points, including 10 research points. According to the clustering results, the differences in functional traits of the two *B. schreberi* clusters were compared using an independent samples *t*-test. Pearson correlation analysis was used to evaluate the relationships between *B. schreberi* functional traits and environmental parameters, as well as the relationships among different functional traits. A random forest model was utilized to analyze the key water and soil factors influencing functional traits. The randomForest [29] package was employed to rank the importance of each trait and environmental factor, with the increase in MSE serving as the criterion to identify the level of contribution. Based on the screening of principal component analysis (PCA), variables with small contributions to the total variance of principal components were removed. The remaining variables were used to construct a structural equation model (SEM) to evaluate the causal relationships between the functional traits of *B. schreberi* and water and soil factors. During the model construction, the functional traits of *B. schreberi* were classified into three categories: morphological, structural, and physiological functions. According to the functional connections of these three types of traits, the influence direction was set as structural parameters affecting morphological expression, and both of them jointly influencing physiological functions. Meanwhile, environmental parameters were divided into two major categories: water and soil parameters. All images in this study were drawn using Adobe Illustrator 2024 and Origin 2024.

## 4. Discussion

### 4.1. Differences Between Growth Stages in the Functional Characteristics of Brasenia schreberi

Stomatal conductance and transpiration rate are generally positively correlated with the photosynthetic rate, because they both reflect the water exchange capacity during photosynthetic carbon assimilation [30]. Compared to sporadic distribution, continuous *B. schreberi* has much higher stomatal conductance, intercellular CO_2_ concentration, transpiration rate, and leaf vein density (Figure 2), indicating stronger photosynthetic water and CO_2_ exchange abilities and thus a potentially higher photosynthetic rate. However, there was no significant difference in the net photosynthetic rate of *B. schreberi* between the two different coverage sites. The water and CO_2_ use efficiencies of plant leaves can be calculated, respectively, through the ratios of net photosynthetic rate to transpiration rate and stomatal conductance. Therefore, the above research results indicate that, compared with sporadically distributed *B. schreberi* (2.11 and 7.81, respectively), continuous distribution reduces the water and CO_2_ use efficiencies of *B. schreberi* (1.66 and 5.37, respectively). That is, achieving the same photosynthetic capacity consumes more water and CO_2_ at a higher coverage [31]. In addition, in sporadic *B. schreberi*, larger aerenchyma and air cavity areas (Figure 2) may enhance air delivery, reducing oxygen stress and channeling more photosynthetic products into growth, thus producing larger leaves [32,33]. A larger leaf area maximizes light harvesting, boosting photosynthesis, water use efficiency, and plant competitiveness [34]. A high coverage of floating leaf plants reduces light penetration and water re-oxygenation, lowers water body oxygen and CO_2_ fixation rates, and thus inhibits photosynthesis [31,35]. An increase in aerenchyma pore area not only enhances short-term air delivery to the lower parts of *B. schreberi* but also boosts CO_2_ uptake, ensuring normal photosynthesis.

### 4.2. Response of Functional Traits of Brasenia schreberi to Aqueous Environmental Factors

Water and soil, key sources of matter and energy for aquatic plant growth, are major environmental factors affecting plant functional traits [36]. The response of *B. schreberi* functional traits to water environment factors confirms our first hypothesis, which is that the growth of *B. schreberi* needs good water quality. Parameters like water pH, dissolved oxygen, and ammonia and nitrate nitrogen, which indicate good water quality, significantly promote the photosynthetic rate and leaf size of *B. schreberi* (Figure S2, Figure 3 and Figure 4, Table 1). For aquatic plants, within a certain pH range (typically between 6.5–7.5), increasing pH can enhance the activity of photosynthetic enzymes like Rubisco, enabling more efficient substrate (e.g., CO_2_) binding and promoting photosynthesis [37]. In addition, within the optimal pH range, aquatic plants have high ion channel permeability in cell membranes, properly functioning carrier proteins, smooth CO_2_ entry into chloroplasts, and efficient O_2_ release, all of which boost photosynthesis and promote larger leaf formation [38,39]. Dissolved oxygen in water significantly boosts the photosynthetic rate and leaf size of *B. schreberi* (Figure S2, Figure 3 and Figure 4, Table 1). When water dissolved oxygen is high, plant roots can respire aerobically, producing adequate ATP that is transported to photosynthetic organs like leaves, ensuring efficient photosynthesis [40]. For floating leaf plants in a high dissolved oxygen environment, the stomata on the plant leaves are more likely to stay open, which not only favors CO_2_ uptake but also lessens the feedback inhibition of photosynthesis caused by oxygen accumulation. Additionally, aquatic plants possess a well-developed system of channels, such as aerenchyma and vascular bundles. In high dissolved oxygen water, plant cells proliferate and enlarge, forming bigger structures like aerenchyma (e.g., the aerenchyma size of *B. schreberi* is positively correlated with water dissolved oxygen) to ensure internal oxygen supply, and larger vascular bundles (with lower vein density) to boost water and nutrient transport [41]. The expansion of these channel structures allows the smooth transport of photosynthetic products to all parts of the aquatic plants, promoting their growth and reproduction.

Nitrogen is a key component of many important biological macromolecules and compounds in plants, such as proteins, nucleic acids, and chlorophyll. Ammonium nitrogen (NH_4_^+^) and nitrate nitrogen (NO_3_^−^) are the two major inorganic nitrogen sources absorbable and utilizable by plants, providing the nitrogen atoms needed for the synthesis of the above-mentioned biomacromolecules [42]. When levels of these two nitrogen forms in water are sufficient, aquatic plants show a marked rise in leaf chlorophyll. This helps plants capture more light energy for the photosynthetic light reaction, supplying ample energy and reducing power. Meanwhile, leaves synthesize more photosynthetic enzymes like Rubisco, accelerating CO_2_ fixation and reducing the Calvin cycle, thus promoting carbon assimilation and boosting the photosynthetic rate [43]. However, excessive NH_4_^+^ concentrations can also be phytotoxic [44], so it is hypothesized that the NH_4_^+^ concentrations in Tengchong Beihai Wetland have not yet reached levels that would harm *B. schreberi*. Although nitrate and ammonium nitrogen promote photosynthetic rate and leaf size in *B. schreberi*, excessively high water total nitrogen and phosphorus levels may lead to opposing results [43,45]. This study also revealed that high total phosphorus levels in water significantly restrict the net photosynthetic rate and leaf size of *B. schreberi*, and there is a significant negative correlation between total nitrogen levels and stomatal conductance (Figure S2, Table 1). Excess nitrogen and phosphorus in water can inhibit a plant’s absorption of other nutrients, causing nutritional imbalance, which in turn weakens the photosynthetic ability and growth potential of the plant. For example, excess phosphorus can lead to excessive accumulation of phosphates in plants, which interferes with zinc uptake [46,47]. *B. schreberi* requires large amounts of zinc to form zinc-rich tissues during growth. Zinc is the main nutrient in its young parts, so restricted zinc absorption may hinder their development. Excessive total nitrogen and phosphorus can also cause eutrophication, leading to algae blooms that consume dissolved oxygen and create hypoxic conditions. This triggers oxidative stress in aquatic plants, generating reactive oxygen species that damage cellular structures and functions, thus inhibiting photosynthesis [48].

High water BOD, COD, and permanganate index also significantly hinder the photosynthetic productivity and leaf growth of *B. schreberi* (Figure S2, Table 1). High water BOD, COD, and permanganate index usually indicate a greater amount of organic pollutants in water. The decomposition of these pollutants consumes a lot of dissolved oxygen, causing a shortage, and releases toxic substances like nitrite, which inhibits photosynthesis and reduces leaf growth and development [45]. High levels of these water parameters can also lead to changes in the physiological metabolism and resource allocation of aquatic plants [49]. Plants may consume significant energy and resources to adjust their antioxidant and defense systems against pollutant toxicity, or reallocate more resources to roots and underground stems, reducing leaf resource investment and affecting their growth and development [49]. The significant positive correlation between the transpiration rate of *B. schreberi* and these parameters (Table 1) may be a response to the restriction of plant photosynthesis in more polluted situations. Plants increase transpiration to improve the supply of water for photosynthesis, thereby maintaining a stable net photosynthetic rate and alleviating the pressure on photosynthetic product requirements for growth and reproduction.

In addition to the impacts of the aforementioned water quality parameters on the functional traits of *B. schreberi*, this study also found that water temperature and depth are significantly negatively correlated with transpiration rate and significantly positively correlated with leaf size (Figure S2, Table 1). Within the suitable temperature range, increased water temperature enhances aquatic plant enzyme activity, benefits photosynthesis, and enables more efficient light energy utilization for organic matter synthesis, thus offering more materials for leaf growth and promoting leaf enlargement [50]. Meanwhile, the increased enzyme activity helps with plant cell division, elongation, and differentiation, and also promotes the growth and enlargement of leaves [50]. However, while rising water temperatures enhance photosynthesis, they can also cause physiological water stress in plants due to excessive water loss from transpiration [51]. In response to physiological water deficit, plants close their stomata to reduce transpiration water loss, which is consistent with previous findings on seagrasses that water temperature affects the balance between photosynthesis and respiration in plants by influencing the photosynthesis irradiance relationship [52]. The impact of water depth on *B. schreberi* may mainly relate to its growth status and interspecific as well as intraspecific competition. In deep waters (>1.5 m) of Tengchong Beihai Wetland, *B. schreberi* is sporadically distributed, with no emergent plants and few submerged plants. Its leaves have enough space to expand, ensuring sufficient water vapour exchange and a photosynthetic rate that meets growth needs. In shallow areas (<1.5 m) of Tengchong Beihai Wetland, *B. schreberi* mainly grows in dense patches competing with submerged plants like bladderwort, horned pondweed, and black algae, leading to intense interspecific and intraspecific competition. In these shallow water areas, *B. schreberi* leaves overlap, so the lower layer leaves cannot obtain enough sunlight, and the plant’s transpiration is also stronger. (Figure 8). To maintain a stable photosynthetic rate, plants increase stomatal conductance and transpiration, but this simultaneous rise reduces water- and CO_2_-use efficiency.

### 4.3. Response of Functional Traits of Brasenia schreberi to Soil Nutrient Conditions

Floating leaf plants primarily acquire nutrients from sediments through their root systems [53], making sediment nutrient conditions crucial in determining plant growth strategies [54,55]. Artificial cultivation of *B. schreberi* shows that its growth requires nutrient-rich soil, especially with abundant organic matter, nitrogen, and phosphorus [17]. However, this study shows that sediment organic carbon, nitrogen, and phosphorus levels are negatively correlated with the net photosynthetic and transpiration rates of *B. schreberi* (Figure 5, Table 1), indicating that the photosynthesis of *B. schreberi* is sensitive to soil nutrient conditions, which inhibit photosynthesis. This finding is a key discovery of this study, although it is in conflict with the second research hypothesis.

Soil is the primary nutrient source for most rooted plants, and its nutrient conditions are a key factor limiting normal plant growth. In most cases, especially in terrestrial habitats, soil nutrients tend to be deficient, and increasing soil nutrients can significantly enhance plant photosynthesis and growth [56,57]. However, wetlands, as nutrient sinks, are frequently subject to eutrophication due to the influx of nutrients like nitrogen and phosphorus. Moderately adding nitrogen and phosphorus to soil can promote chlorophyll synthesis in aquatic plant leaves and increase stomatal opening, thereby raising photosynthetic and transpiration rates [58]. However, excessive soil nitrogen and phosphorus not only cause nitrogen waste and pollution but also negatively affect plant nutrition, stomatal conductance, and photosynthetic efficiency, ultimately reducing the net photosynthetic rate [59]. The south, north, and west of Tengchong Beihai Wetland are farmland and villages, and the east is a major tourist spot. With constant nutrient input from these sources, the soil nutrient levels in the wetland are already high. The main distribution location of *B. schreberi* in Tengchong Beihai Wetland is in the wetland restoration area in the south of the wetland. Long-term farming has made the soil in these areas fertile, with high carbon (135.05 g kg^−1^), nitrogen (9.17 g kg^−1^), and phosphorus (1.29 g kg^−1^) content. These levels are significantly higher than in other *B. schreberi* production areas, such as the soil in Hunan Chaling (total nitrogen: 5.05 g kg^−1^, total phosphorus: 0.82 g kg^−1^, organic matter: 205.00 g kg^−1^) [60]. Consequently, it can be inferred that the existing soil nutrient levels in Tengchong Beihai Wetland are sufficiently high to sustain the robust growth of *B. schreberi*. Any additional increase in soil nutrients would surpass the optimal tolerance threshold of this species, thereby resulting in suboptimal growth.

Although most morphological parameters are not significantly related to sediment element content, soil total phosphorus significantly impacts stomatal density and size. Stomatal density shows a significant positive correlation with soil total phosphorus, while stomatal size (length, width, area) exhibits a significant negative correlation (Figure S2, Figure 3 and Figure 4, Table 1). This indicates that stomatal morphological parameters are sensitive to soil phosphorus content, implying that soil phosphorus affects stomatal function. Stomata are the main pathways for water and CO_2_ exchange in plants and the primary route for water loss [61,62]. Under conditions of high soil fertility, the increase in stomatal density may enhance water vapor exchange, thereby compensating for the decrease in photosynthetic and transpiration rates and maintaining the stability of leaf photosynthesis. A high phosphorus environment may promote epidermal cell proliferation via the cytokinin signaling pathway (e.g., ARR12—mediated cell proliferation), raising stomatal density per unit leaf area [47]. There exists a certain trade-off relationship between stomatal size and density. As stomatal density escalates, stomatal size tends to diminish correspondingly in order to preserve normal stomatal function and maintain leaf structural integrity [63]. Overly large stomata may compromise the stability of the leaf internal architecture, thereby disrupting the normal physiological processes [63]. Smaller stomata are more efficient at reducing water loss when closed, thus enhancing plant water-use efficiency [64]. In the context of elevated soil phosphorus levels, plants may preferentially augment stomatal density to boost photosynthetic efficiency while concurrently reducing stomatal size to optimize water use efficiency, thereby attaining a balance between growth and water management [64].

Other soil nutrients, such as meso- and micro-elements, are also crucial for plant growth, as they can influence photosynthesis by modulating one or more steps in plant physiological processes [65]. For instance, potassium can boost root growth, helping roots absorb more water and nutrients from deeper soil layers and promoting the plant’s vertical growth [66]. Calcium, a vital cell wall component, binds with pectic substances to strengthen cell wall mechanical strength and promote cell splitting and expansion [66]. Magnesium, a component of chlorophyll and a cofactor for many enzymes, directly engages in respiration and photosynthesis. It also regulates the absorption and utilization of potassium, calcium, nitrogen, etc., thus promoting plant growth [67]. Zinc, as a cofactor of carbonic anhydrase, catalyzes the hydration of CO_2_ to form HCO_3_^−^, indirectly boosting CO_2_ availability around Rubisco and enhancing photosynthesis. Silicon deposited in plant cell walls as silicified walls enhances leaf mechanical strength, adjusts stomatal opening and closing sensitivity, and thus boosts transpiration rate. Furthermore, silicon can form silica polymers within leaf cells that alleviate damage to photosynthetic structures caused by environmental stress, thereby maintaining normal photosynthetic function [68]. In this study, the net photosynthetic and transpiration rates of *B. schreberi* generally showed a positive correlation with soil levels of potassium, magnesium, zinc, and silicon, and a negative correlation with soil calcium levels (Figure S2, Table 1). This indicates that potassium, magnesium, zinc, and silicon enhance photosynthetic productivity either by directly participating in the photosynthetic processes of *B. schreberi* or by promoting chlorophyll synthesis. Furthermore, more calcium helps *B. schreberi* enhance its mechanical strength. This is a plant adaptation to adversity, implying that under unfavorable conditions, photosynthesis weakens, and more photosynthetic products are consumed.

**Figure 10:**
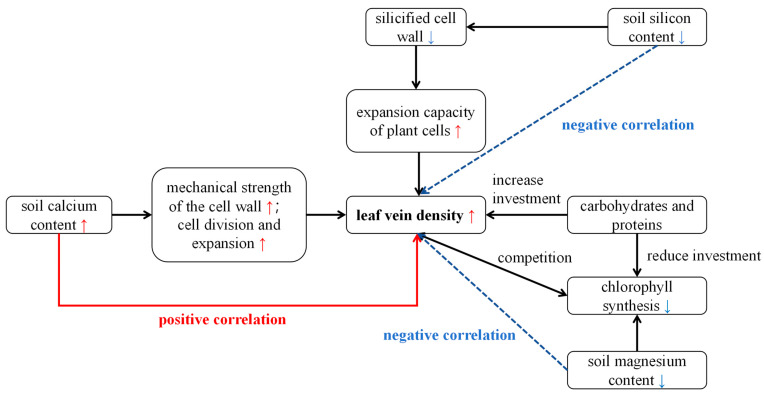
The relationship between vein density and soil nutrients. Red arrows indicate positive correlations, while blue arrows indicate negative correlations.

Unlike photosynthetic parameters, vein density shows a significantly positive correlation with soil calcium and negative correlations with magnesium and silicon content (Figure S2, Table 1). Veins are key transport channels for water, nutrients, and photosynthetic products in plants and play a critical role in ensuring the stability of photosynthesis [69]. In addition to providing structural support for leaves and keeping them spread out, veins require carbohydrates and proteins for their development. This may create competition with chlorophyll synthesis, so when magnesium levels rise, vein density can decrease [69]. Silicon from sediments precipitates in plant mechanical tissues as silicified cell walls, which may restrict vein expansion and reduce vein density [70]. Conversely, calcium may enhance cell wall mechanical strength, promoting cell division and expansion, and thus increase vein construction and stability. The response of veins to soil nutrients (Figure 10) also indicates the degree of adversity that *B. schreberi* faces.

### 4.4. Functional Linkages Among Brasenia schreberi Traits

During responses to water and soil environmental changes, *B. schreberi* functional traits exhibit certain functional linkages. Many studies have indicated a positive correlation between net photosynthetic rate, stomatal conductance, and transpiration rate. This study also found the same relationships in *B. schreberi* (Figure 5 and Figure 6), reflecting the dependence of photosynthesis on stomatal water and CO_2_ conductance. Stomata reflect the trade-off between plant H_2_O protection and CO_2_ capture [71]. High stomatal conductance means strong water conduction, higher transpiration rates, and ample CO_2_ supply for photosynthesis, thus supporting higher photosynthetic rates. As photosynthesis utilizes more CO_2_, the intercellular CO_2_ concentration decreases. Additionally, net photosynthetic rate correlates positively with leaf size, stomatal size, and pore size (Figure 5 and Figure 6), aligning with previous studies [35]. Larger leaves often mean a bigger photosynthetic area, allowing for more light absorption and higher photosynthetic efficiency. Stomata, which facilitate gas exchange by letting CO_2_ in and releasing oxygen and water vapor, directly impact intercellular CO_2_ concentration and, consequently, photosynthesis [72]. Larger stomata can supply more CO_2_, enhancing photosynthesis. However, they often have lower opening/closing sensitivity [72,73]. This means that under stress, plants may suffer greater photosynthetic damage, partly explaining *B. schreberi*’s endangered status. Aquatic plants can fill tissues with air via aerenchyma pores to increase buoyancy. This helps them float and reduces hypoxia stress. Larger aerenchyma pores enhance breathing and photosynthetic activity [32,74]. The three traits—leaf size, stomatal size, and aerenchyma pore size—in *B. schreberi*, due to their similarly positive effects on photosynthetic function, are significantly functionally coordinated.

## 5. Conclusions

The functional traits of *B. schreberi* are closely related to its growth patterns. Compared to sporadically distributed individuals, plants in continuous patches exhibit higher stomatal conductance, transpiration rate, and vein density, but significantly smaller leaf size, reduced aerenchyma area, and diminished aerial pore area. This indicates that when *B. schreberi* grows too densely, its water and CO_2_ utilization efficiency, leaf size, and gas transport ability decrease, while transport efficiency increases. The photosynthetic productivity and leaf size of *B. schreberi* are positively correlated with water temperature, dissolved oxygen, nitrate, and ammonium nitrogen in water, and negatively correlated with total nitrogen, total phosphorus, BOD, COD, and permanganate index. This indicates that *B. schreberi* requires good water quality for growth. The photosynthetic productivity of *B. schreberi* shows a significantly negative correlation with soil organic carbon, nitrogen, and phosphorus, but a significantly positive correlation with soil magnesium, zinc, and silicon. This indicates that, while *B. schreberi* requires certain micronutrients for optimal growth, its demand for major soil nutrients like carbon, nitrogen, and phosphorus is somewhat limited. The soil in Tengchong Beihai Wetland is fertile and rich in nutrients. Adding more soil nutrients can be harmful to *B. schreberi*. When adapting to water and soil changes, there are functional links among its functional traits. Stomatal conduction of water and CO_2_, leaf light harvesting, and aerenchyma gas transmission are functionally linked. Enhanced performance in these processes can significantly boost *B. schreberi*’s photosynthetic carbon assimilation. These findings demonstrate that alterations in aquatic environments and soil conditions significantly impact the growth and survival of *B. schreberi*. Specifically, water quality degradation coupled with eutrophication of water and soil may impair the photosynthetic productivity of *B. schreberi* and reduce its efficiency in water and CO_2_ utilization, ultimately leading to suboptimal growth or even extinction of this species. These findings offer physiological explanations for the endangered mechanisms of *B. schreberi* and are highly significant for its scientific conservation and utilization. Based on the results of this study, it is necessary to pay special attention to the content of nutrients in water and soil, especially the input of nitrogen and phosphorus, in the conservation and management of *B. schreberi*. In addition, appropriately increasing the content of trace elements such as magnesium, zinc, and silicon in the soil can play a certain role in improving the photosynthetic productivity of *B. schreberi*. When carrying out habitat restoration and species reintroduction, improving water quality and reducing the content of the three main soil nutrients, carbon, nitrogen, and phosphorus, are the keys to the success of reintroduction.

## Figures and Tables

**Table 1:** Correlations between leaf functional traits of *Brasenia schreberi* and environmental parameters.

Parameter	WH	WT	PH	DO	N_water_	P_water_	NH_4_^+^	NO_3_^−^	BOD	COD	COD_Mn_	C_soil_	N_soil_	P_soil_	K	Ca	Mg	Zn	Si
P_n_	−0.114	−0.101	0.434 **	0.315 **	−0.211	−0.292 *	0.328 **	0.485 **	−0.127	−0.165	−0.060	−0.370 **	−0.384 **	−0.360 **	0.214	−0.223	0.314 **	0.366 **	0.362 **
G_s_	−0.091	−0.216	−0.037	−0.144	−0.277 *	−0.021	−0.017	−0.180	−0.034	−0.069	−0.053	−0.033	−0.060	0.18	0.068	0.057	0.043	0.110	−0.108
C_i_	−0.063	0.025	−0.423 **	−0.312 **	0.038	0.280 *	−0.335 **	−0.477 **	0.218	0.246 *	0.163	0.090	0.106	0.210	0.076	0.007	−0.059	−0.011	−0.145
T_r_	−0.435 **	−0.407 **	0.049	−0.214	−0.165	−0.063	−0.139	0.153	0.270 *	0.241 *	0.300 *	−0.353 **	−0.396 **	−0.308 *	0.345 **	−0.288 *	0.236	0.431 **	0.259 *
LA	0.407 **	0.436 **	0.441 **	0.664 **	−0.184	−0.315 **	0.330 **	0.285 *	−0.397 **	−0.363 **	−0.405 **	0.128	0.191	0.028	−0.237	0.031	−0.043	−0.104	0.007
LP	0.375 **	0.434 **	0.409 **	0.663 **	−0.177	−0.297*	0.379 **	0.292 *	−0.342 **	−0.317 **	−0.351 **	0.169	0.232	0.011	−0.251*	0.046	−0.057	−0.122	−0.001
L/W	0.277 *	0.190	0.079	0.309 *	−0.141	−0.106	−0.031	−0.108	−0.219	−0.187	−0.258	0.040	0.047	0.029	−0.123	−0.009	−0.086	−0.034	−0.042
LP^2^/LA	0.014	0.104	0.015	0.186	−0.036	−0.028	0.260 *	0.114	0.109	0.083	0.102	0.194	0.207	−0.078	−0.129	0.079	−0.095	−0.118	−0.033
SD	0.086	0.294	−0.020	0.165	−0.106	0.054	0.055	−0.059	−0.062	−0.039	−0.026	0.139	0.218	0.374 **	−0.122	0.168	−0.031	−0.176	−0.188
SL	0.049	−0.114	0.348 **	0.113	0.078	−0.08	0.262 *	0.348 **	0.040	−0.012	0.026	0.060	0.006	−0.361**	−0.104	−0.017	−0.103	−0.057	0.119
SW	−0.069	−0.161	0.039	0.017	−0.006	−0.13	0.139	0.190	0.142	0.098	0.132	−0.036	−0.086	−0.283*	0.056	−0.063	−0.029	0.031	0.120
SA	−0.009	−0.152	0.184	0.06	0.037	−0.104	0.197	0.261 *	0.096	0.045	0.080	0.008	−0.051	−0.339 **	−0.022	−0.044	−0.071	−0.011	0.123
VD	−0.047	−0.354 **	−0.139	−0.433 **	−0.184	0.039	−0.235	−0.256 *	0.029	−0.011	0.011	0.201	0.136	0.182	−0.107	0.355 **	−0.339 **	−0.216	−0.366 **
BA	−0.044	0.069	−0.119	−0.127	−0.054	−0.146	0.155	−0.045	−0.030	−0.050	−0.009	−0.011	−0.005	−0.009	0.174	0.060	0.049	−0.012	0.062
AS	0.067	0.114	0.029	0.095	0.196	−0.051	0.006	0.103	0.170	0.190	0.125	0.079	0.095	−0.169	−0.066	−0.069	−0.065	−0.049	0.093
AA	0.122	0.110	0.071	0.244 *	−0.231	−0.371 **	0.158	0.129	−0.095	−0.076	−0.087	−0.089	−0.070	−0.225	0.090	−0.091	0.134	0.110	0.226
CT	−0.099	−0.352 **	0.100	−0.161	−0.209	−0.153	−0.044	0.129	0.010	−0.023	0.034	0.068	0.021	−0.015	−0.056	0.104	−0.179	−0.106	−0.141
ET	−0.054	0.168	−0.045	0.21	−0.037	−0.299 *	0.260 *	0.156	−0.073	−0.072	−0.057	−0.148	−0.101	−0.028	0.218	−0.116	0.209	0.186	0.176

*, *p* < 0.05; **, *p* < 0.01. Bold font indicates a significant difference. P_n_, net photosynthetic rate; G_s_, stomatal conductance; C_i_, intercellular carbon dioxide concentration; T_r_, transpiration rate; LA, leaf area; LP, leaf perimeter; L/W, ratio of leaf length to width; LP^2^/LA, ratio of square of leaf perimeter to area; SD, stomatal density; SL, stomatal length; SW, stomatal width; SA, stomatal area; VD, leaf vein density; BA, vascular bundle area; AS, air space area; AA, spiracle area; CT, cuticle thickness; ET, epidermal thickness; WH, water height; WT, water temperature; PH, acidity and alkalinity; DO, dissolved oxygen; N_water_, total nitrogen; P_water_, total phosphorus; NH_4_^+^, ammonia nitrogen; NO_3_^−^, nitrate nitrogen; BOD, biological oxygen demand; COD, chemical oxygen demand; COD_Mn_, potassium permanganate; C_soil_, total soil carbon; N_soil_, total soil nitrogen; P_soil_, total soil phosphorus; K, soil potassium mass fraction; Ca, soil calcium mass fraction; Mg, soil magnesium mass fraction; Zn, soil zinc mass fraction; Si, soil silicon mass fraction.

## Data Availability

The data that support the findings of this study are available from the corresponding author upon reasonable request. Please contact the author at sm0510215@163.com.

## Supplementary Materials

The following supporting information can be downloaded at: https://www.mdpi.com/article/10.3390/plants14132072/s1, Figure S1. Workflow diagram of anatomical and morphological characteristics. Figure S2. Randomforest modeling of functional traits of *B. schreberi* leaves in relation to water and substrate. Photosythetics parameters in relation to water and substrate(a); form parameters in relation to water and substrate(b); structure parameters in relation to water and substrate(c). P_n_, net photosynthetic rate; G_s_, stomatal conductance; C_i_, intercellular carbon dioxide concentration; T_r_, transpiration rate; LA, leaf area; LP, leaf perimeter; L/W, ratio of leaf length to width; LP^2^/LA, ratio of square of leaf perimeter to area; SD, stomatal density; SL, stomatal length; SW, stomatal width; SA, stomatal area; VD, leaf vein density; BA, vascular bundle area; AS, air space area; AA, spiracle area; CT, cuticle thickness; ET, epidermal thickness; WH, water height; WT, water temperature; PH, acidity and alkalinity; DO, dissolved oxygen; Nwater, total nitrogen; Pwater, total phosphorus; NH_4_^+^, ammonia nitrogen; NO_3_^−^, nitrate nitrogen; BOD, biological oxygen demand; COD, chemical oxygen demand; COD_Mn_, potassium permanganate; C_soil_, total soil carbon; N_soil_, total soil nitrogen; P_soil_, total soil phosphorus; K, soil potassium mass fraction; Ca, soil calcium mass fraction; Mg, soil magnesium mass fraction; Zn, soil zinc mass fraction; Si, soil silicon mass fraction.
