# Supplementary material for: Functional Trait Responses of Brasenia schreberi to Water and Soil Conditions Reveal Its Endangered Status
Source: Plants (Basel). 2025 Jul 7;14(13):2072. doi: 10.3390/plants14132072 (PMC12252049; doi:10.3390/plants14132072)
Supplement: Supplementary file 1 [file plants-14-02072-s001.zip › Figure S2.pdf]

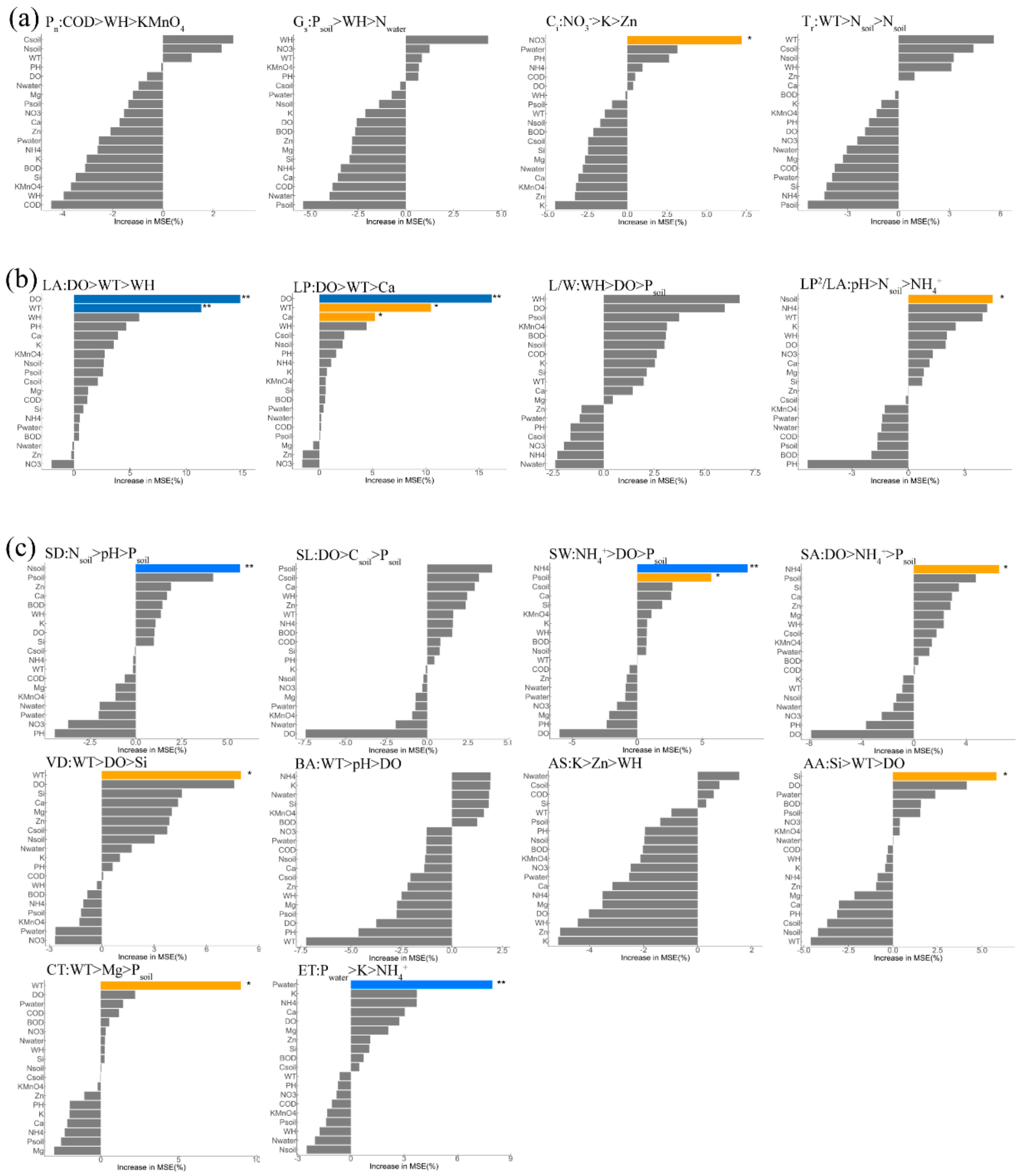

Figure S2. Randomforest modeling of functional traits of *B. schreberi* leaves in relation to water and substrate. Photosythetics parameters in relation to water and substrate(a); form parameters in relation to water and substrate(b); structure parameters in relation to water and substrate(c). P_n, net photosynthetic rate; G_s, stomatal conductance; C_i, intercellular carbon dioxide concentration; T_r, transpiration rate; LA, leaf area; LP, leaf perimeter; L/W, ratio of leaf length to width; LP^2/LA, ratio of square of leaf perimeter to area; SD, stomatal density; SL, stomatal length; SW, stomatal width; SA, stomatal area; VD, leaf vein density; BA, vascular bundle area; AS, air space area; AA, spiracle area; CT, cuticle thickness; ET, epidermal thickness; WH, water height; WT, water temperature; PH, acidity and alkalinity; DO, dissolved oxygen; N_{water}, total nitrogen; P_{water}, total phosphorus; NH_4^+, ammonia nitrogen; NO_3^-, nitrate nitrogen; BOD, biological oxygen demand; COD, chemical oxygen demand;

COD_{Mn}, potassium permanganate; C_{soil}, total soil carbon; N_{soil}, total soil nitrogen; P_{soil}, total soil phosphorus; K, soil potassium mass fraction; Ca, soil calcium mass fraction; Mg, soil magnesium mass fraction; Zn, soil zinc mass fraction; Si, soil silicon mass fraction.
