# Supplementary figures and images for: Functional Trait Responses of Brasenia schreberi to Water and Soil Conditions Reveal Its Endangered Status
Source: Plants (Basel). 2025 Jul 7;14(13):2072. doi: 10.3390/plants14132072 (PMC12252049; doi:10.3390/plants14132072)

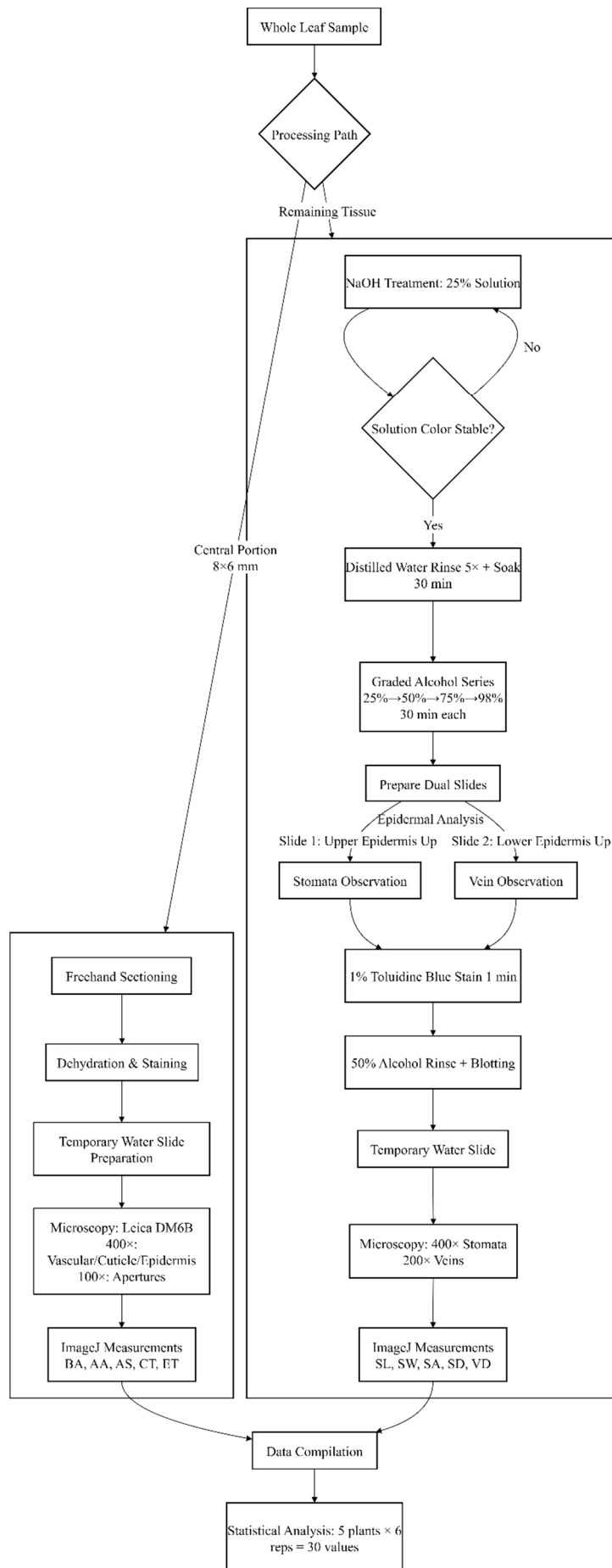

Figure S1. Workflow diagram of anatomical and morphological characteristics.

Supplement: Supplementary file 1 [file plants-14-02072-s001.zip › Figure S1.pdf]
